# A quick and cost effective method for the diagnosis of *Mycobacterium ulcerans *infection

**DOI:** 10.1186/1471-2334-12-8

**Published:** 2012-01-18

**Authors:** Dziedzom K de Souza, Charles Quaye, Lydia Mosi, Phyllis Addo, Daniel A Boakye

**Affiliations:** 1 Parasitology Department, Noguchi Memorial Institute for Medical Research, University of Ghana, P. O. Box, LG 581, Legon-Accra, Ghana; 2 Department of Biochemistry, University of Ghana, Legon, Ghana; 3 Animal Experimentation Department, Noguchi Memorial Institute for Medical Research, University of Ghana, Legon, Ghana

**Keywords:** Buruli ulcer, *Mycobacterium ulcerans*, Diagnosis, Loop mediated isothermal amplification

## Abstract

**Background:**

Buruli ulcer (BU), a neglected tropical skin disease caused by *Mycobacterium ulcerans*, has been reported in over 30 countries worldwide and is highly endemic in rural West and Central Africa. The mode of transmission remains unknown and treatment is the only alternative to disease control. Early and effective treatment to prevent the morbid effects of the disease depends on early diagnosis; however, current diagnosis based on clinical presentation and microscopy has to be confirmed by PCR and other tests in reference laboratories. As such confirmed BU diagnosis is either late, inefficient, time consuming or very expensive, and there is the need for an early diagnosis tool at point of care facilities. In this paper we report on a simple, quick and inexpensive diagnostic test that could be used at point of care facilities, in resource-poor settings.

**Methods:**

The methodology employed is based on the loop mediated isothermal amplification (LAMP) technique. Four sets of Primers, targeting the mycolactone encoding plasmid genome sequence of *M. ulcerans *were designed. The BU-LAMP assay was developed and tested on five *M. ulcerans *strains from patients in Ghana and two American Type Culture Control (ATCC) reference isolates; Ghana #970321 (D19F9) and Benin #990826 (D27D14). We also tested the assay on other closely related, mycolactone-producing mycobacterial strains; *M. marinum *1218, *M. marinum *DL240490, *M. liflandii *and *M. pseudoshotsii*, as well as experimentally infected laboratory animal and clinical samples.

**Results:**

The results revealed a high specificity of the BU-LAMP assay for selectively detecting *M. ulcerans*. Compared to the conventional IS-2404 PCR, the new assay is cheaper and simpler and ten times more sensitive. Test results can be obtained within 1 hour.

**Conclusions:**

This study indicates that the BU-LAMP assay could be suitable for early disease diagnosis and application in low-resource health facilities.

## Background

*Mycobacterium ulcerans*, a bacterium belonging to the same family as *M. tuberculosis *and *M. leprae*, is the causative agent of Buruli ulcer (BU). BU has been described as a neglected tropical disease and is the third most common mycobacterial infection after tuberculosis and leprosy [1]. It is a necrotizing, painless, cutaneous infection mainly localized on the limbs of affected individuals and causes extensive damage to the skin, its associated tissues and even the bone. The pathology of the disease is due to mycolactone, a necrotizing and immunosuppressive lipid toxin produced by the bacterium. The genes that code for the production of the toxin are located on a 174 kb plasmid. In endemic countries like Ghana, Cote d'Ivoire and Benin, the disease is prevalent in rural poor communities. In the most endemic district in Ghana a prevalence of up to 150.8 per 100,000 individuals has been reported [2]. However epidemiological studies suggest underreporting and improper diagnosis as some of the factors hindering the determination of the exact disease burden in endemic areas. In all endemic areas, the infection burden has long term socio-economic impacts on infected individuals and their communities.

Current reporting of cases of BU infection is based on the presentation of the symptoms. This represents a challenge due to the vast number of other skin infections or conditions that may exhibit symptoms similar to that of BU [3-5]. The WHO has therefore directed that all clinically diagnosed or suspected cases of BU be confirmed. Currently, this can only be done in reference laboratories, since the current methods are not amenable to point of care diagnosis.

Laboratory confirmation of BU is complex and has evolved over the years. *Mycobacterium ulcerans *stains red (acid-fast bacilli, AFB) in the Ziehl-Neelsen staining procedure but this method has a low sensitivity [6]. Swabs taken from lesions often do not show AFB by microscopic examination. Culturing *M*. *ulcerans *from clinical samples is difficult and has a low sensitivity of about 35-60% [6,7]. The bacterium is notoriously slow-growing (6-8 weeks) and culture media are frequently contaminated with other faster growing species [8]. PCR methods have been developed for BU diagnosis based on the 16S rRNA gene [6], the *hsp65 *gene [9], or the insertion sequence IS-*2404 *[10]. Although the sensitivity of PCR is high (98%), this method is expensive and requires technical expertise in terms of DNA extraction and equipment needed.

Notwithstanding the fact that a combination of these methods can lead to an accurate diagnosis, the highly technical and expensive nature of the techniques confines them to reference laboratories. Thus, there is no simple, rapid test that is appropriate for early point of care diagnosis in the low-resourced laboratory settings where the disease is most prevalent. This represents a huge gap between early diagnosis before ulcers and deformities occur and the critical need for treatment and prevention of associated deformities. We report the development of a simple and relatively inexpensive test for *M. ulcerans *diagnosis, which could easily be applied in basic healthcare facilities, without recourse to expensive, complex and time-consuming methods. This new method is based on a novel DNA amplification method, developed by Notomi and colleagues [11].

## Methods

The methodology used is termed loop mediated isothermal amplification (LAMP) technique and has been applied for the molecular diagnosis of a variety of diseases including; *P. falciparum *malaria, West Nile Virus, Influenza, Tuberculosis, Trypanosomiasis and filarial detection in mosquitoes [12-16].

### Primer design

The mycolactone encoding plasmid genome sequence, *pMUM001*, of *M. ulcerans Agy99 *(GenBank accession no. BX649209.1) was compared with *M. marinum *1218 (GenBank accession no. EU271967.1); primers were designed from regions with the lowest sequence similarity, using the LAMP primer design software, PrimerExplorer V4 http://primerexplorer.jp/e/. Four sets of primers targeting different regions of pMUM001 were generated.

### Preparation of templates

The bacterial strains used in this study are listed in Table 1. We also used tissue biopsy samples obtained from lesions of mice that had been experimentally infected with *M. ulcerans*. Finally, the assay was tested on archived/stored human samples (Table 2). The Noguchi Memorial Institute for Medical Research is one of three reference centers in Ghana where BU samples from patients are sent for confirmation. For this study, no samples were directly taken from patients; the assay was only tested on stored samples. The DNA template from these samples was prepared by boiling and Qiagen extraction. For the boiling method, each sample was ground in a mortar, transferred to an eppendorf tube and incubated in a water-bath at 90°C for 20 minutes. The extraction of DNA, using the Qiagen kit, followed the recommended procedures from the manufacturers. The DNA concentration for the samples extracted using the Qiagen kit was quantified by measuring the optical density at 260 nm.

**Table 1:** Mycobacteria strains

Strain	Species	Host	Geographic origin	Reference or source
D19F9	*M. ulcerans*	Human	Ghana	ATCC 97031

D27D14	*M. ulcerans*	Human	Benin	ATCC 990826

-	*M. ulcerans*	Human	Ghana	This paper

-	*M. ulcerans*	Human	Ghana	This paper

-	*M. ulcerans*	Human	Ghana	This paper

-	*M. ulcerans*	Human	Ghana	This paper

-	*M. ulcerans*	Human	Ghana	This paper

1218	*M. marinum*	Salt water fish	United states marine captive	ATCC 927

DL240490	*M. marinum*	Sea Bass (Dicentrarchus labrax)	Red Sea Israel	[17]

L15	*M. pseudoshotsii*	Striped bass (Morone saxatilis)	Chesapeake Bay	[17]

XL5	*M. liflandii*	Frog (Xenopus laevis)	University of Virginia	[17]

**Table 2:** Results of BU-LAMP assay test on Human samples

Sample ID	Sample type	Date collected	LAMP Assay		PCR
				
			Boil Prepared DNA	Qiagen extracted DNA	
1A	Swab in no medium	-	***	***	***

1B	Swab in no medium	April 2010	***	***	***

1C	Swab in no medium	Feb 2010	**Positive**	**Positive**	**Positive**

1D	Swab in no medium	April 2010	**Positive**	**Positive**	**Positive**

1E	Swab in no medium	-	***	***	***

2A	Tissue in medium	April 2010	***	***	***

2E	Tissue in medium	April 2010	***	**Positive**	***

2F	Tissue in medium	April 2010	**Positive**	**Positive**	***

2W	Tissue in medium	April 2010	***	**Positive**	***

2X	Tissue in medium	April 2010	***	**Positive**	***

2B	Swab in medium	Feb 2010	***	***	***

2C	Swab in medium	April 2010	***	***	***

2D	Swab in medium	April 2010	***	**Positive**	**Positive**

2G	Swab in medium	April 2010	***	***	***

2I	Swab in medium	April 2010	***	**Positive**	***

3A	Fine needle aspirate	April 2010	***	***	***

3B	Fine needle aspirate	-	***	**Positive**	***

3C	Fine needle aspirate	-	**Positive**	**Positive**	***

3E	Fine needle aspirate	-	**Positive**	**Positive**	***

4A	Punch biopsy	-	**Positive**	**Positive**	***

4B	Punch biopsy	-	***	***	***

4C	Punch biopsy	-	***	**Positive**	***

***No amplification observed

### LAMP assay

The BU-LAMP assay protocol was optimized for maximum efficiency, using the Loopamp DNA amplification kit (Eiken Chemical), and performed according to the manufacturer's protocol. LAMP assays were performed in 25 μl reactions each containing 20 μM of each inner primer (FIP and BIP), 5 μM of each outer primer (F3 and B3), 20 μM each of the loop primers (FLP and BLP), 12.5 μl of reaction mix, 1 μl of fluorescent detection reagent, 1 μl of *Bst *DNA polymerase and 2 μl of prepared DNA templates. The reaction mixture was incubated at 65°C for 60 min followed by an enzyme inactivation step of 90°C for 10 min. Optimization was carried out using either a thermal cycler or a water bath. The use of the latter was to ensure the applicability of the LAMP assay in poorly resourced health facilities. The assay was tested in duplicate on DNA templates prepared from all samples as described above. Products were visualized under UV light directly in the eppendorf tubes. A positive and a negative control were included in both conventional PCR and BU-LAMP reactions. Sensitivity testing was based on 100-fold serial dilutions up to 10^-8^. For the purpose of practical applicability in low-resourced laboratories, the dilution beyond which no discernable differences between the samples and the negative control could be detected under UV light (by the naked eye) was considered the sensitivity limit of the assay. Determination for positivity was done visually, by gel electrophoresis on 1.5% gels stained with ethidium bromide, or by measuring the optical density at 350 nm or 450 nm.

### Polymerase chain reaction

In order to compare the specificity and sensitivity of the LAMP assay, all samples were subjected to conventional PCR. We used current PCR method for diagnosis and confirmation of *M. ulcerans *infection, targeting the IS2404 gene [10]. Briefly, 5 μl of DNA template was amplified in 25 μl reactions using a buffer supplied by the manufacturer of *Taq *polymerase (Promega), 1.5 mM MgCl2, 1 mM of each primers, 200 mM (each) deoxynucleoside triphosphates, and 1 U of *Taq *polymerase. The reactions were performed in an automated thermal cycler (MJ Research), using an initial denaturation of 94°C for 2 min, followed by 35 cycles of 1 minute steps at 94°C, 66°C, and 72°C, and a final elongation step of 72°C for 7 minutes. PCR amplicons were visualized by gel electrophoresis on 1.5% gels stained with ethidium bromide.

### Ethical approval

Ethical approval for the use of animal and archived/stored human samples was obtained from the Institutional Review Board of the Noguchi Memorial Institute for Medical Research.

## Results

### BU-LAMP primer for *M. ulcerans *detection

**Figure 1:**
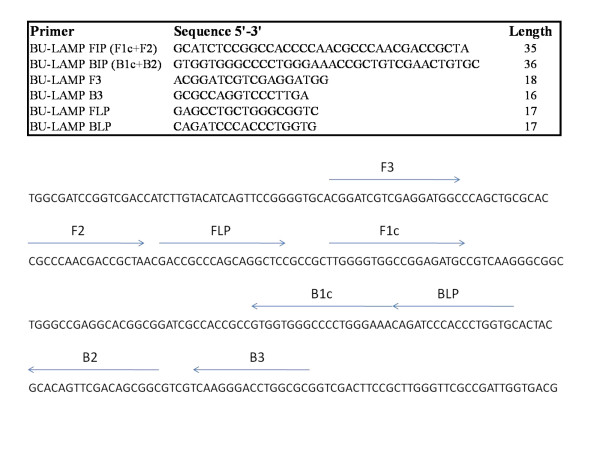
**BU-LAMP primer arrangements and sequences**.

Primers were designed from positions 7303 to 7483 of the *M. ulcerans *plasmid sequence. A set of 6 primers, consisting of two outer (F3 and B3), two inner (FIP and BIP) and two loop primers (FLP and BLP) were designed. The primer sequences and arrangements are shown in Figure 1. Primers FIP and BIP are the combinations of 2 sequences consisting F1c and F2 and B1c and B2 respectively. These primers amplify a 180 bp fragment.

### BU-LAMP assays

The BU-LAMP assay was successful, amplifying *M. ulcerans *at 65°C. Amplification results could also be observed after incubating for 15 minutes. Trials incubated in both a thermal-cycler and the water-bath gave similar results.

**Figure 2:**
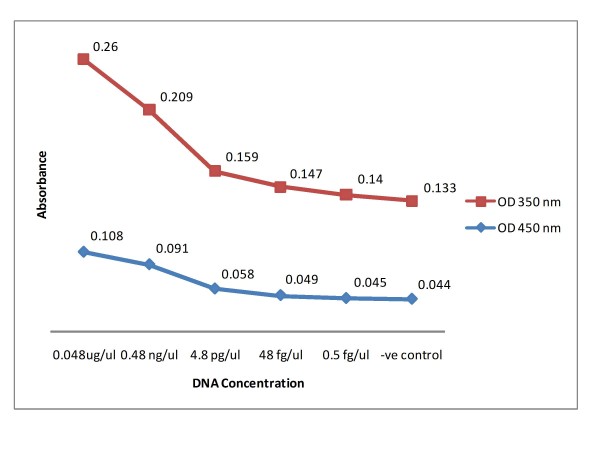
**Absorbance-concentration curve of BU-LAMP products**.

Tests performed on the very closely related *M. marinum *1218 and other mycolactone producing mycobacteria; *M. marinum *DL240490, *M. liflandii *and *M. pseudoshotsii *were negative indicating the BU-LAMP primers to be specific to *M. ulcerans*. The DNA concentration at the visual detection limit was estimated to be 48 pg/μl. However, by measuring the OD value of BU-LAMP products (OD values measured at 350 and 450 nm) we were able to detect positivity when using DNA concentrations as low as 0.5 fg/μl (Figure 2).

### Tests on animal and human samples

**Figure 3:**
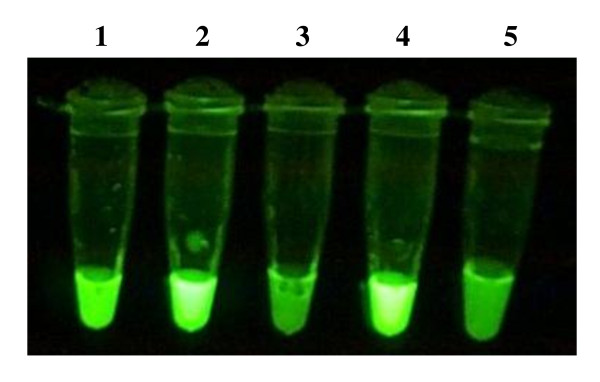
**Visualization of BU-LAMP products exposed to UV light**. Tube 1 contains DNA extracted using the boil preparation method; tube 2 contains Qiagen extracted DNA; tube 3 contains raw/untreated sample; tube 4 is the positive control and tube 5 is the negative control.

**Figure 4:**
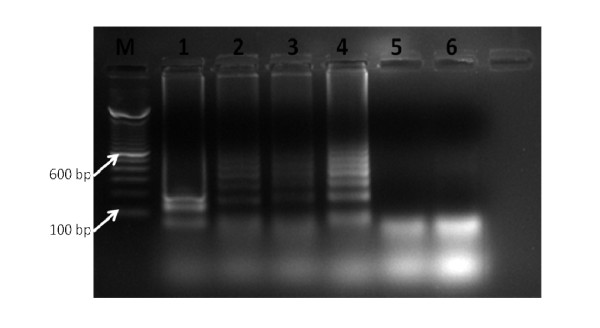
**Gel electrophoregram of BU-LAMP products**. M = 100 base pair marker, Lane 1 = raw sample, Lane 2 = Boil prepared DNA, Lane 3 = Qiagen extracted DNA, Lane 4 = Positive control, Lane 5 = *M. marinum *DNA, Lane 6 = Negative control.

9.5 μg of pure DNA was obtained from approximately 50 mg of mice biopsy sample, using the Qiagen DNA extraction method. Both the Qiagen-extracted and boil-prepared DNA produced results, with the former giving the best results (Figure 3). Raw samples, i.e. macerated samples without heat or chemical treatment, used in the assay failed to produce visibly-detectable change in color intensity when examined directly under UV light (Figure 3). However, running the BU-LAMP products from raw samples on an agarose gel revealed them to produce amplifications (Figure 4).

Results of the BU-LAMP test on clinical samples are shown in Table 2. Samples were tested twice for the confirmation of the results. Thirteen of the 22 samples were positive for the Qiagen extracted DNA, while only 6 samples were positive for the boil- prepared DNA, when tested using the BU-LAMP method. On the other hand, only 3 of the samples tested positive using the conventional PCR method. Also, samples collected using fine needle aspirates (3/4), punch biopsy (2/3) and tissue stored in growth medium (4/5), gave the highest positivity compared to swabs stored or not stored in medium.

## Discussion and conclusions

The diagnosis of BU relies primarily on culture [8] and, conventional and real-time PCR methods [6,10,18,19]. However, these methods are time consuming, expensive and limited to well-resourced laboratories. The BU-LAMP assay on the other hand is amenable to point of care diagnosis in rural health facilities, due to the ease, simplicity and low technical expertise required.

The BU-LAMP assay specifically amplified DNA from *M. ulcerans *but not from the closely related *M. marinum *or any of the other *Mycobacterium *species tested. These results clearly demonstrate the high specificity of the BU-LAMP assay in detecting *M. ulcerans*, compared to other methods, such as the IS-2404 PCR [10,20], which amplifies other environmental mycobacteria, especially those that produce mycolactone [21,22]. This new assay also has a very high sensitivity, as it proved to be 10 times more sensitive than the most used PCR method, based on IS-2404 [10,20]. We noted that the high level of sensitivity of the LAMP assay at amplifying minute amounts of DNA was one of the things that needed fine tuning in subsequent research, particularly for adaptation to point of care facilities.

The evaluation of the BU-LAMP method in animal biopsy samples showed successful amplification of DNA by the Qiagen extraction and boil preparation methods, and the use of raw samples. However, the failure of raw samples in producing visibly-detectable results may be because there was not enough DNA that could be amplified, or due to the presence of inhibitors, which may have been inactivated through heat incubation (boil preparation) or the pure Qiagen DNA extraction method. Nonetheless, the fact that heat-treated samples produced visibly detectable results is of significance for the applicability of this method in resource-poor settings. The use of heat-treated samples in low-resource settings eliminates the requirements for time-consuming and expensive DNA extraction methods. The time involved from DNA extraction to result was considerably lower, since the BU-LAMP results can be obtained within 1.5 hours compared to about 6 hours for the conventional PCR. In addition, costing of this new assay was estimated at US$ 4 per sample, compared to the conventional PCR method of approximately US$ 6.0 per sample. The assay also eliminates the use of thermal-cyclers and expensive UV illuminators. The need for UV lights can be removed completely through the use of SYBR green dye [23].

Of the human samples analyzed, 59% and 27% were positive using the BU-LAMP method on Qiagen and boil-prepared- DNA respectively. Only 14% of samples were positive using the conventional PCR method on Qiagen extracted DNA. The discrepancy between BU-LAMP positive and PCR positive results could be attributed to the high sensitivity of the BU-LAMP method and its ability to detect DNA quantities 10 times lower than the sensitivity limit of 0.1 *M. ulcerans *genome equivalent established for the IS-2404 method [20]. These results may have been affected by various factors such as the time from sample collection to processing, the medium of storage and the reliability of the collections. The samples tested had been in storage for at least 5 months; therefore, future implementation research studies should focus on analyzing samples immediately after they have been collected. Although the total sample size was small, fine needle aspirates, punch biopsies and tissue in medium provided the best results and may be considered as the best sample types for field application of the BU-LAMP assay. The extent of disease will also have to be considered in the acquisition of samples. Despite all the possible limitations and the low number of samples analyzed, the results show that in our study the LAMP method performed better than the conventional PCR method.

In conclusion, the BU-LAMP method shows promise as a diagnostic tool at point of care facilities in BU endemic communities. This new method requires very little manipulation of BU samples, and amplifies DNA with high specificity and rapidity under constant temperature conditions (in a water bath). Unlike the conventional PCR method that requires the use of a thermal cycler, purified DNA samples are not a requirement. DNA amplification occurs within 1 hour and the resulting product is a turbid solution, indicative of product amplification. Sample confirmation can therefore be done visually with the naked eye, and the intensity of the fluorescence observed is indicative of amount of DNA present. In addition to these characteristics, other basic requirements such as cold storage facility for reagents and water bath are usually available at point of care facilities and therefore make the BU-LAMP method feasible as a point of care test. However, there is the need for further studies to determine the sensitivity of the BU-LAMP assay, on a larger sample size, of freshly collected sample types (skin biopsy, punch biopsy, swabs and fine needle aspirates), and on the efficacy and applicability of the assay in low-resourced laboratory settings.

## Competing interests

The authors declare that they have no competing interests.

## Authors' contributions

DKS conceived the study, participated in the study design, the experiments and drafting of the manuscript. CQ and LM designed and performed the experiments and drafted the manuscript. PA provided *M. ulcerans *strains and animal biopsy samples and drafted the manuscript. DAB designed the study and drafted the manuscript. All authors read and approved the final manuscript.

## Pre-publication history

The pre-publication history for this paper can be accessed here:

http://www.biomedcentral.com/1471-2334/12/8/prepub
